# Genome editing in the treatment of ocular diseases

**DOI:** 10.1038/s12276-023-01057-2

**Published:** 2023-08-01

**Authors:** Elliot H. Choi, Susie Suh, Avery E. Sears, Rafał Hołubowicz, Sanjay R. Kedhar, Andrew W. Browne, Krzysztof Palczewski

**Affiliations:** 1 Gavin Herbert Eye Institute, Department of Ophthalmology, University of California, Irvine, CA USA; 2 Department of Physiology and Biophysics, University of California, Irvine, CA USA; 3 Department of Chemistry, University of California, Irvine, CA USA; 4 Department of Molecular Biology and Biochemistry, University of California, Irvine, CA USA

**Keywords:** Targeted gene repair, Targeted gene repair, Genetic engineering

## Abstract

Genome-editing technologies have ushered in a new era in gene therapy, providing novel therapeutic strategies for a wide range of diseases, including both genetic and nongenetic ocular diseases. These technologies offer new hope for patients suffering from previously untreatable conditions. The unique anatomical and physiological features of the eye, including its immune-privileged status, size, and compartmentalized structure, provide an optimal environment for the application of these cutting-edge technologies. Moreover, the development of various delivery methods has facilitated the efficient and targeted administration of genome engineering tools designed to correct specific ocular tissues. Additionally, advancements in noninvasive ocular imaging techniques and electroretinography have enabled real-time monitoring of therapeutic efficacy and safety. Herein, we discuss the discovery and development of genome-editing technologies, their application to ocular diseases from the anterior segment to the posterior segment, current limitations encountered in translating these technologies into clinical practice, and ongoing research endeavors aimed at overcoming these challenges.

## Eye disease: gene editing offers new treatment possibilities

Using gene editing tools to correct gene mutations and manipulate specific tissues in the eye could revolutionize treatment for multiple eye diseases. Krzysztof Palczewski and co-workers at the University of California in Irvine, USA, reviewed the current status of gene editing tools such as CRISPR-Cas9 for eye diseases. The eye’s special ‘immune privilege’ status means it can tolerate therapeutic interventions without excessive inflammatory responses. The eye is also easy to image for monitoring. Recent successful trials on mouse models include gene editing treatments for glaucoma and inherited retinal diseases. Initial human trials using CRISPR-Cas9 to treat an eye condition triggered by the herpes simplex virus showed promise. The latest generation of gene editing tools and delivery systems are increasingly precise, limiting unwanted side effects. However, challenges remain in bringing this technology into clinical practice.

## Introduction

Genome-editing technologies have evolved as powerful tools for the precise modification of DNA sequences within living organisms, offering new therapeutic avenues for a wide range of diseases, both genetic and nongenetic in etiology^1,2^. The most well-known and widely used genome-editing technology is the clustered regularly interspaced short palindromic repeats and CRISPR-associated protein 9 (CRISPR‒Cas9) system, derived from the bacterial adaptive immune system. The CRISPR‒Cas9 system has been used for targeted genome editing due to its simplicity, precision, and versatility^3–5^. In addition to CRISPR‒Cas9, newer technologies, including base editors and prime editors, have been developed to further expand the capabilities and precision of genome editing^6–8^. Base editors enable precise conversion of one nucleotide to another without generating double-strand breaks (DSBs) in the target DNA. Prime editors offer a versatile genome-editing platform that can introduce a broad range of desired alterations, including targeted insertions, deletions, and all 12 types of point mutations, without necessitating DSBs or donor DNA templates^8^. These advanced genome-editing technologies have been instrumental in advancing our understanding of the genetic basis of diseases and hold great promise for the development of novel therapeutic interventions^5,9–11^.

Ocular diseases encompass a wide range of conditions that can significantly impact vision and overall quality of life. Conventional treatment options aim to alleviate symptoms or delay disease progression, and in some cases, surgical interventions are necessary. However, these approaches are not always curative, and many patients still experience substantial vision loss despite the best available treatments. Genome editing holds promise in revolutionizing ocular treatment, offering new avenues for patients with currently limited options^12–15^. The unique characteristics of the eye, including its immune-privileged status, small size, and compartmentalized structure, facilitate the efficient delivery and maintenance of genome-edited components without eliciting excessive immune responses. This allows local administration of therapeutic agents and minimizes the risk of systemic exposure. Additionally, the eye can be evaluated by noninvasive imaging techniques, such as optical coherence tomography, fundoscopy, angiography, and the newer generation of two-photon microscopy, facilitating real-time monitoring of therapeutic outcomes and safety^16–20^. By directly targeting underlying genetic causes or modulating gene expression to address nongenetic etiologies, these innovative technologies have the potential to prevent vision loss in numerous individuals. However, as the field continues to advance, it is crucial to evaluate the efficacy, safety, and applicability of genome editing in the treatment of ocular diseases while also considering the ethical implications of these technologies.

In this review, we discuss the discovery and development of CRISPR‒Cas9 systems, base editors, and prime editors and their applications in the treatment of ocular diseases in the sequence of ocular anatomy from the anterior segment to the posterior segment. Furthermore, we address the current limitations of these technologies, including issues related to off-target effects, efficacy, immunogenicity, and delivery methods. Finally, we discuss ongoing research aimed at addressing these challenges.

## Genome-editing technologies

### Discovery and development of CRISPR‒Cas systems for genome editing

CRISPR, initially identified in the 1980s, serves as a component of the bacterial immune system. CRISPR was first observed in *Escherichia coli* when repetitive DNA sequences interspaced with unique spacers were discovered^21^. Subsequent investigations revealed that these sequences were present in approximately 40% of sequenced bacterial genomes and 90% of archaeal genomes, suggesting a conserved function across prokaryotes^22–24^. Further studies showed that the unique spacer sequences originated from foreign DNA, such as plasmids and bacteriophages^25,26^, leading to the hypothesis that these sequences play a crucial role in the adaptive immune response of prokaryotes, protecting them against invading genetic elements. In support of this hypothesis, it was shown that the presence of a spacer sequence matching a particular bacteriophage in the CRISPR locus correlated with resistance to that phage, while the absence of such a matching sequence conferred susceptibility^27^. CRISPR-associated (Cas) genes, located adjacent to CRISPR loci, were found to encode proteins that facilitate the immune response by targeting and degrading invading nucleic acids^28^.

### Conventional CRISPR‒Cas9 nuclease

**Fig. 1:**
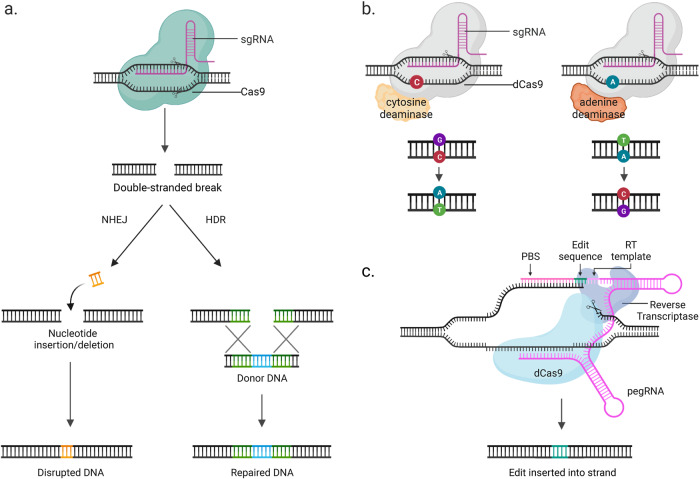
CRISPR‒Cas9-based genome-editing approaches. **a** The CRISPR‒Cas9 system can introduce double-stranded breaks (DSBs) in target DNA. Cells have two repair mechanisms: nonhomologous end-joining (NHEJ) and homology-directed repair (HDR). NHEJ rejoins the cleaved ends of DNA, resulting in deletions or insertions. On the other hand, HDR relies on a template for repair. The template can be donor DNA or a sister chromatid, which is used as a template to copy the correct sequence into the cleaved ends. **b** Cytosine base editors (CBEs) and adenine base editors (ABEs) are genome-editing tools that introduce specific nucleotide changes without generating DSBs. CBEs result in a C•G to T•A conversion, and ABEs result in an A•T to G•C conversion. **c** Prime editors (PEs) utilize a Cas9 nickase fused with reverse transcriptase to introduce new DNA sequences at the target locus without generating DSBs. PegRNA, an extended sgRNA containing the template sequence for reverse transcription, directs nucleotide synthesis at the target locus.

CRISPR‒Cas systems have emerged as powerful tools for genome editing due to their simplicity, efficiency, and versatility. CRISPR‒Cas systems can be classified into three main types (Types I, II, and III), distinguished by the presence of specific Cas proteins encoded adjacent to the CRISPR array^29^. Among these types, Type II systems, which include the Cas9 endonuclease, have been utilized frequently because they require only a single protein to perform diverse functions^30^. A transformative breakthrough in CRISPR technology came in 2012 when the use of CRISPR‒Cas9 for genome editing in mammalian cells was demonstrated, which sparked a wave of interest in the field^3,31^. The CRISPR‒Cas9 system consists of two main components: a single-guide RNA (sgRNA) that targets a specific DNA sequence and the Cas9 protein, which functions as a molecular scissor to cut DNA at the targeted location, resulting in a DSB (Fig. 1a). Following the introduction of a DSB by Cas9, the endogenous repair machinery utilizes one of two major pathways to repair the break: nonhomologous end-joining (NHEJ) or homology-directed repair (HDR)^32^. NHEJ is the pathway most commonly used by CRISPR‒Cas9 for genome editing. When Cas9 cuts the DNA strand, the repair machinery is activated and attempts to rejoin the broken ends through NHEJ. This process can introduce small insertions or deletions (indels) at the cut site, which can disrupt the function of the targeted gene. This makes NHEJ well suited for applications where the goal is to disrupt or knock out a gene, as it can efficiently introduce frameshift mutations that often result in a loss of function.

HDR, on the other hand, is a more precise and controlled mechanism for genome editing^33^. In this pathway, a donor template containing the desired genetic modification is introduced into the cell along with the Cas9 and an sgRNA^34^. The repair machinery then uses this template to repair the DNA at the cut site, resulting in a precise modification of the genome^34^. The ratio of NHEJ to HDR can vary widely depending on several factors, including the cell type, the delivery method of the CRISPR‒Cas9 system, and the specific experimental conditions used^35^. In general, NHEJ is more prevalent than HDR, and this imbalance can often limit the efficiency of HDR-mediated genome editing. In some cell types, the ratio of NHEJ to HDR has been reported to be as high as 100:1, while in others, the ratio is closer to 10:1^36^. Moreover, HDR occurs less frequently than NHEJ in eukaryotic cells or postmitotic cells, making precise genome editing more challenging^32^.

While conventional CRISPR‒Cas9 has been widely used for genome editing, it has some limitations in terms of its efficiency and precision. To address these issues, new genome-editing tools have been developed, such as base editors and prime editors^6,8,10,37–40^. These tools provide a more precise and efficient way to edit the genome, and they hold great promise for therapeutic application. In the following paragraphs, we discuss the principles of base editors and prime editors and their advantages over conventional CRISPR‒Cas9^6,8,10,37–40^.

### Base editors and variants

Introduced in 2016, base editors have emerged as a promising genome-editing tool that addresses the limitations associated with conventional CRISPR‒Cas9^6^. Base editors enable precise installation of target point mutations without DSBs, as they consist of a catalytically impaired Cas9 nuclease, often Cas9 nickase (nCas), and a deaminase enzyme (Fig. 1b). Upon nCas binding to the corresponding DNA site, the provided sgRNA hybridizes with the target DNA strand, displacing the genomic DNA strand and resulting in a single-stranded DNA loop structure known as an R-loop. This process allows the protospacer adjacent motif (PAM) to become accessible to the deaminase domain of the base editor^41,42^. Base editors are classified into two main types: cytosine base editors (CBEs) and adenine base editors (ABEs). CBEs utilize cytidine deaminases to convert cytosine (C) to uracil (U), which is subsequently recognized as thymine by the repair machinery^6,43,44^. ABEs employ laboratory-evolved adenosine deaminases to convert adenine (A) to inosine (I), which is then recognized as guanine by the repair machinery^7^. Consequently, CBEs and ABEs facilitate targeted base conversion from C•G to T•A and A•T to G•C, respectively.

Compared to Cas nucleases, base editors exhibit significantly higher efficiency for the desired editing with a lower incidence of indels or undesired byproducts of DSBs^8,39,45^. The development of base editors has facilitated the study of specific point mutations and their effects on gene function and pathogenesis. Moreover, base editors have shown promise in various therapeutic applications, including correcting pathogenic mutations or modulating gene expression by targeting regulatory elements^10,46–52^. Since the first introduction of base editors, numerous efforts have been made to enhance their precision and efficiency, leading to the development of several variants.

In 2017, the fourth-generation CBE, also referred to as BE4, was developed by using a new uracil glycosylase inhibitor domain and a smaller Cas9 protein, significantly reducing off-target mutations and increasing specificity and efficiency^53^. Shortly thereafter, modifications to the nuclear localization sequence and ancestral reconstruction of the deaminase component yielded CBE and ABE variants, named BE4max and ABEmax, with up to six- and sevenfold improvements in editing efficiency, respectively^38,54^. In 2020, phage-assisted noncontinuous evolution (PANCE) and continuous evolution (PACE) and bacterial selection methods were applied to improve the efficiency and compatibility of the original ABEs (ABE7.10)^55,56^. PANCE and PACE are powerful strategies that enable self-directed evolution in bacteria, thereby improving the activity of a target protein variant in significantly shorter timespans^56^. The evolved ABE variant, ABE8e, contains eight additional mutations in the deaminase domain and exhibits a 590-fold increase in activity compared to ABE7.10^55^. These new variants have also substantially expanded the scope of targetable mutations by using various homologs of Cas9 or Cas12, which can recognize different PAMs^57^.

Although base editors, with continuous development and improvements, hold great potential for the treatment of monogenic diseases, they do have some limitations. First, base editors are unable to perform certain types of DNA modifications. While they can correct six out of the 12 possible single-nucleotide substitutions, base editors cannot perform all 12 possible base conversions, nor can they implement small sequence insertions or deletions in the genome. These limitations restrict the scope of targetable mutations. Second, despite the high degree of precision exhibited by base editors, off-target effects and bystander editing can still occur. Bystander editing is a phenomenon in which nontargeted neighboring DNA sequences are edited, potentially leading to unintended consequences and raising safety concerns^58,59^. Last, despite the development of various PAM-compatible base editors, not all DNA sequences are targetable with currently available variants. Addressing these limitations will be crucial for ensuring the safe and effective therapeutic application of base editors.

### Prime editors and different versions

Prime editors (PEs), first described in 2019, are the latest generation of genome-editing tools that address several limitations of prior genome-editing approaches^8^. Unlike base editors, prime editors can perform a wide range of edits, including insertions, deletions, and all 12 types of single-base substitutions, without relying on DSBs or donor DNA templates. This technology substantially expands the capacity to correct up to 89% of known pathogenic human mutations^8^. Moreover, prime editing has been shown to have a lower risk of off-target effects and bystander editing than base editing^60^.

Prime editors consist of a fusion protein that combines a Cas9 H840A nickase with a reverse transcriptase (RT) and a prime-editing guide RNA (pegRNA) (Fig. 1c). The pegRNA contains a 20-nucleotide protospacer sequence, a primer binding site, and an RT template that encodes the desired DNA sequence. Upon binding to the target DNA, the Cas9 nickase nicks the nontargeted DNA strand, while the RT copies the desired edit from the pegRNA template to synthesize a new DNA strand. The repair machinery then replaces the original DNA strand with the newly synthesized strand, incorporating genetic modifications with higher precision and fewer undesired byproducts than conventional CRISPR‒Cas9 systems.

The editing process with prime editors necessitates three checkpoints of complementary base pairing: hybridization between the pegRNA spacer and the target locus, hybridization between the pegRNA primer binding site and the nicked 3’ end of the target DNA, and hybridization between the reverse-transcribed 3’ DNA flap and the downstream genomic sequence. Due to the requirement for multiple checkpoints of complementary base pairing, prime editors exhibit high specificity and a low rate of off-target effects^8,60,61^. Additionally, because prime editing uses an RT to copy the edited DNA sequence from the pegRNA template, the editing process is localized to the immediate vicinity of the target site, minimizing the likelihood of bystander effects.

Numerous efforts have been devoted to refining prime editors for enhanced efficiency and specificity, yielding PE2 and PE3 from the original prime editor, PE1^8^. PE2 demonstrates improved fidelity and editing efficacy by introducing five mutations that increase the enzymatic activity of the RT. PE3 incorporates an additional sgRNA to nick the nonedited DNA strand. This approach promotes preferential repair of the edited strand, leading to an increase in editing efficiency up to 4.1-fold compared to PE2, albeit with a higher percentage of indels^8^. Additional strategies to refine prime editors include engineering the prime editor protein^62,63^, improving pegRNA stability and structure^64,65^, using two pegRNAs^66^, improving the accessibility of the target DNA^61,67^, and circumventing DNA mismatch repair^68^. These approaches have led to the development of numerous prime-editor variants. For instance, the latest generation prime editors, PE4 and PE5, enhance the editing outcome by manipulating cellular repair-mechanism pathways. DNA mismatch repair (MMR) has been shown to impede prime editing and promote undesired mutant byproducts. By expressing an engineered MMR-inhibiting protein, PE4 and PE5 enhance the editing efficiency by up to 7.7-fold and 2.0-fold compared to PE2 and PE3, respectively, while reducing the indel byproducts^68^.

In conclusion, prime editors represent a major advancement in the field of genome editing, offering the ability to perform a wide range of precise edits with fewer undesired byproducts and a lower risk of off-target effects. The ongoing evolution of prime editors further enhances their efficiency and specificity. These improvements in prime editor technology hold great promise for the treatment of genetic diseases and the advancement of biotechnology. However, further research and development are essential to fully harness the potential of prime editors and to ensure their safe and effective use in clinical applications.

## Application of genome editing in the treatment of ocular diseases

The human eye is a complex and essential organ that plays a pivotal role in perceiving light, color, and depth to facilitate daily activities and interactions. The human eye consists of various interconnected cell types, ranging from the corneal epithelium in the anterior chamber of the eye to the retinal ganglion cells (RGCs) that project to the brain. In particular, photoreceptors, which are responsible for the initiation of the visual signaling cascade, and retinal pigment epithelial (RPE) cells, which support the function of photoreceptors and contribute to visual chromophore regeneration, play key roles in detecting light and transmitting visual information to the brain^69^. Vision loss can occur as a result of disruptions to these cells, caused by either pathogenic mutations or environmental factors, significantly impacting the quality of life^70^. Approximately 2.2 billion individuals worldwide are affected by visual impairments, underscoring the need for innovative treatments for both inherited and nongenetic ocular disorders^71^.

The eye possesses several distinctive features that render it an ideal candidate for gene therapy and genome-editing approaches. First, its immune privilege status allows it to tolerate foreign molecules and therapeutic agents without eliciting strong inflammatory or immune responses, which is crucial for the success of gene therapy approaches^72,73^. Second, the small size, compartmentalization, and easy accessibility facilitate the delivery and localization of therapeutic agents^74^. Last, the anatomical location and transparent nature of the lens enable noninvasive, real-time monitoring of treatment outcomes, which greatly aids in assessing the efficacy and safety of novel therapies. Consequently, inherited retinal diseases (IRDs) have become a primary focus of gene-augmentation therapy, RNA-targeting therapy, and genome editing^75–77^.

A significant milestone in ocular gene therapy was achieved when the US Food and Drug Administration (FDA) approved voretigene neparvovec (Luxturna) in 2017 to treat Leber congenital amaurosis (LCA), caused by mutations in the *RPE65* gene^78^. Luxturna employs adeno-associated virus (AAV) vectors to deliver a functional *RPE65* gene into the RPE^78^. While Luxturna has shown promise in restoring vision for patients with LCA, treated individuals have often experienced a continuation of retinal degeneration and relapse in visual acuity a few years after treatment^79–82^. Moreover, the application of Luxturna is limited to patients with specific mutations, and Luxturna would not be suitable for patients with ocular disorders caused by other mutations. Subsequent advances in genome-editing technologies, including the development of CRISPR/Cas9, base editors, and prime editors, now hold promise for addressing a broader range of inherited and nongenetic ocular disorders^13,14^. In the following section, we discuss the progress in applying genome-editing technologies to various ocular disease models, as well as the genotypic or phenotypic effects and potential clinical implications of these advancements.

### Corneal diseases

The cornea is a transparent, avascular, and dome-shaped structure that covers the anterior surface of the eye^83^. The cornea plays a critical role in refracting and focusing light onto the retina, which is essential for clear vision^83^. This refractive power of the cornea accounts for approximately two-thirds of the total refractive power of the eye^84^. Additionally, the cornea serves as a structural barrier and protects the eye against pathogens and other foreign materials. The cornea consists of five layers, the epithelium, Bowman’s layer, the stroma, Descemet’s membrane, and the endothelium, each with distinct functions and properties^83^.

The cornea is prone to various disorders and diseases that can arise from a variety of causes, including infections, genetic mutations, or autoimmune disorders. For instance, herpetic stromal keratitis (HSK) is caused by the herpes simplex virus and can lead to inflammation, corneal scarring, and vision loss if left untreated^85^. Fuchs endothelial corneal dystrophy (FECD), on the other hand, is a genetic disorder that impacts the endothelial cells of the cornea, causing swelling, clouding, and decreased vision^86^. Granular corneal dystrophy (GCD) is another genetic disorder that affects the stromal layer of the cornea, resulting in the accumulation of protein deposits and reduced vision^87^. These diseases can significantly impact a patient’s quality of life, and innovative treatments are required to prevent progression and restore vision.

#### Herpetic stromal keratitis

Herpes simplex virus type 1 (HSV-1) is a major cause of infectious blindness, with no treatment currently available to eliminate the virus from the infection site or from latent reservoirs in the trigeminal ganglia^88^. A recent study demonstrated that HSV-1-erasing lentiviral particles (HELP) can effectively target two genes of HSV-1 that are essential for viral replication, UL8 and UL29, through the delivery of *Streptococcus pyogenes* Cas9 (SpCas9) mRNA and targeting sgRNAs^89–91^. Intrastromal administration of HELP efficiently blocked HSV-1 replication and the occurrence of HSK in three different animal models^91^. Additionally, HELP was capable of eliminating the viral reservoir via retrograde transport from the cornea to the trigeminal ganglia. Importantly, HELP effectively eliminated HSV-1 in human corneal tissue culture without off-target effects, suggesting its potential as an effective antiviral therapy for HSK. These results are encouraging, as HELP can be administered to patients with acute corneal perforation or corneal graft failure due to the recurrence of the virus^91^. The high treatment efficacy in animals and relative safety of mRNA-based therapy could accelerate the clinical translation of HELP^91^. HELP was evaluated in three patients with severe refractory herpes stromal keratitis during corneal transplantation, with an average follow-up of 18 months^92^. In two patients, HSV-1 DNA became undetectable immediately after the treatment. In a patient with a higher viral load, HSV-1 DNA became undetectable 6 months after the treatment. No off-target effects or Cas9 and vector-related immune responses were observed in these patients. Overall, these studies provide valuable insights into the potential of CRISPR‒Cas9 as a therapeutic tool for HSK. Future studies comparing HELP with conventional acyclovir treatment will be critical to fully evaluate its efficacy and safety.

#### Fuchs endothelial corneal dystrophy

FECD is a progressive ocular disorder affecting corneal endothelial cells, and it can lead to impaired vision and, in severe cases, the need for corneal transplantation^86^. A missense mutation in the collagen type VIII alpha 2 chain (*COL8A2*) gene has been identified as a cause of early-onset FECD^93,94^. Although other mutations in zinc finger E box-binding homeobox 1 (*ZEB1*) and trinucleotide expansion in transcription factor 4 (*TCF4*) are strongly associated with FECD, only the mouse model harboring the *Col8a2* missense mutation recapitulated the features of FECD^95,96^. A recent study demonstrated the potential of CRISPR‒Cas9-mediated gene editing as a therapeutic approach to target the *Col8a2* mutation in the early-onset FECD mouse model. A single anterior-chamber injection of an adenovirus encoding SpCas9 and sgRNA targeting the start codon of *Col8a2* was reported to effectively knock out mutant COL8A2 protein expression in corneal endothelial cells^97^. Ten months after the injection, treated eyes exhibited significant improvements in corneal endothelial cell density and a reduction in the formation of guttae-like structures compared to untreated eyes^97^. Furthermore, this approach rescued endothelium-pumping function in a mouse model^97^. These results suggest that the reduction in mutant COL8A2 protein levels with CRIPSR-Cas9 may have therapeutic potential in treating or extending the survival of corneal endothelial cells in early-onset FECD, potentially circumventing the need for transplantation.

#### Granular corneal dystrophy

GCD represents a group of rare, inherited corneal diseases characterized by progressive vision loss due to the accumulation of granular deposits in the stromal layer of the cornea. Two main clinical types exist: GCD1 and GCD2. GCD2, also referred to as Avellino corneal dystrophy, arises from the R124H mutation in the transforming growth factor-beta induced (*TGFBI*) gene^87,98^. Patients with GCD2 develop granular deposits in the corneal stroma at an early age, and as they grow, older amyloid deposits appear in the deeper stroma^99,100^. These deposits can cause recurrent corneal erosions, corneal opacity, and a subsequent decrease in visual acuity. Disease progression of GCD2 varies between heterozygous and homozygous GCD2 patients, with homozygous individuals typically experiencing onset before the age of 10 years and more rapid progression^101,102^. When recurrent corneal erosions persist despite conservative management, surgical interventions such as photorefractive keratectomy or keratoplasty could be considered^103,104^. However, these treatments are often associated with recurrence and complications. Given these limitations, the pursuit of alternative therapies has become a significant area of interest. A recent study employed CRISPR‒Cas9 and a 100-nucleotide donor template to correct the R124H mutation in primary corneal keratocytes from a GCD2 patient^105^. Among the analyzed clones, 20.6% exhibited monoallelic correction, and 41.3% showed biallelic correction^105^. Consequently, 62% of clones showed successful R124 allele correction derived from the donor template. Although this strategy has not yet been evaluated in vivo, the results suggest that CRISPR‒Cas9-mediated gene correction could be a therapeutic strategy for GCD2.

### Glaucoma

Primary open-angle glaucoma (POAG) is a complex, chronic, and progressive optic neuropathy characterized by the gradual degeneration of retinal ganglion cells. This degeneration results in a thinning of the retinal nerve fiber layer, optic disc cupping, and ultimately irreversible vision loss^106^. POAG is the most prevalent form of glaucoma, accounting for approximately 74% of all glaucoma cases worldwide^107^. The etiology of POAG is multifactorial, involving a combination of genetic, physiological, and environmental factors. Notably, elevated intraocular pressure (IOP) is a major risk factor, mainly attributed to the imbalance between aqueous humor production and outflow^106,108^. Moreover, genetic factors can contribute to elevated IOP in POAG. Specifically, mutations in the myocilin (*MYOC*) gene represent the leading genetic cause, accounting for 4% of POAG cases and 30 to 40% of adult-onset juvenile glaucoma cases^109^. The mutant Y437H-MYOC proteins are misfolded and accumulate in the endoplasmic reticulum (ER), causing chronic ER stress and trabecular meshwork (TM) cell dysfunction or death^110,111^. Given the toxic gain of function, targeting and reducing the expression of the mutant MYOC protein represents a promising therapeutic strategy for POAG^112^.

A recent study demonstrated the effectiveness of CRISPR‒Cas9 in treating a POAG mouse model expressing the mutant Y437H-MYOC^112^. Intravitreal injection of adenovirus carrying Cas9 and sgRNA achieved 60–70% transduction efficiency of TM cells in mice. When mice were treated before one month of age, IOP elevation, ER stress, and subsequent glaucomatous damage were mitigated. Moreover, the treatment improved both TM-cell function and RGC function, as measured by pattern electroretinography. The study further assessed the impact of CRISPR‒Cas9 in ex vivo perfusion-cultured human eyes, demonstrating that this approach could effectively reduce mutant-MYOC expression and alleviate ER stress in human TM cells. These findings highlight the translational potential of CRISPR‒Cas9 genome editing for the treatment of *MYOC* mutation-related POAG in patients. However, considering that glaucoma often arises from a combination of genetic and environmental factors and is rarely monogenic, the development of a gene therapy capable of effectively lowering IOP and addressing the needs of a broader POAG population remains a critical goal.

To address this challenge, a versatile gene therapy that reduces IOP by decreasing aqueous humor production has been developed. This therapy involves a single intravitreal injection of AAV carrying SpCas9 and sgRNA targeting exon 1 of Aquaporin 1 (*Aqp1*) in mouse ciliary-body epithelium^113^. AQP1 is a water-channel protein expressed in various tissues, including the ciliary-body epithelium, where it plays a crucial role in the regulation of aqueous humor production. The altered expression and function of AQP1 have been implicated in the pathophysiology of glaucoma, suggesting its potential as a therapeutic target for glaucoma. CRISPR‒Cas9 targeting *Aqp1* was shown to lead to a reduction in IOP in treated eyes (10.4 ± 2.4 mmHg) compared to control eyes (13.2 ± 2.0 mmHg)^113^. Applied in both the corticosteroid-induced mouse model of ocular hypertension and the microbead-glaucoma mouse model, eyes treated with this approach showed decreased IOP and the loss of fewer ganglion cells compared with untreated eyes. Importantly, this approach also produced detectable indel formation in the *AQP1* locus of ex vivo cultured human ciliary epithelium. The decrease in IOP resulting from disrupted AQP1 expression is similar to that which occurs with existing treatments, including carbonic anhydrase inhibitors, beta-adrenergic receptor blockers, and alpha-adrenergic receptor agonists. A significant advantage of this approach is that it is a one-time treatment, which eliminates patient-compliance issues related to daily eye drop administration.

Another gene associated with POAG is transforming growth factor-beta 2 (*TGF*β*2*), which encodes a multifunctional cytokine involved in extracellular matrix production, cell proliferation, differentiation, and migration^114^. Among TGFβ isoforms in the eye, TGFβ2 is predominant and found in large amounts in the aqueous humor of the anterior segment^115–117^. Emerging evidence has revealed a significant association between TGFβ2 and the pathogenesis of glaucoma. Elevated levels of TGFβ2 in the aqueous humor and TM have been identified in patients with POAG^118–120^. Likewise, the optic nerve heads of patients with POAG appear to contain 70- to 100-fold higher amounts of TGFβ2 than those of age-matched control subjects^121^. The precise mechanism by which elevated TGFβ2 contributes to the pathogenesis of glaucoma remains unknown. Furthermore, no mutations in *TGF*β*2* or its receptor causing POAG have been identified^122^. Consequently, gene correction or disruption targeting *TGF*β*2* is not suitable for treating POAG. However, epigenetic modifications, such as the deacetylation of the *TGF*β*2* gene promoter, have been proposed to lower TGFβ2 levels and IOP in POAG.

In pursuit of this goal, in a recent study, the CRISPR interference system was utilized to selectively deacetylate histones in the *TGF*β*2* gene promoter, subsequently leading to a decrease in TGFβ2 levels^122^. CRISPR interference utilized a catalytically dead Cas9, known as dCas9, which sterically hinders the binding of RNA polymerase or interferes with the transcription elongation process, thereby inhibiting transcription. In this study, dCas9 fused with the Kruppel-associated box domain (KRAB), which functions as a histone deacetylase, was used to enhance transcriptional repression^123,124^. KRAB-dCas9 and sgRNA lowered TGFβ2 levels in cultured TM cells and tissues and ameliorated ocular hypertension in a TGFβ2-overexpressing mouse model^122^. These findings demonstrated that epigenetic editing with the CRISPR interference system holds promise for advancing the development of innovative therapeutics.

### Pathologic neovascularization

Neovascularization, the process of new blood vessel formation, is essential for normal tissue growth, repair, and wound healing^125^. However, aberrant neovascularization contributes to various ocular diseases, including exudative age-related macular degeneration (AMD), diabetic retinopathy, retinopathy of prematurity, and other conditions resulting in ischemic retinal vasculopathy^126–129^. In these conditions, the uncontrolled growth of abnormal blood vessels threatens normal vision and may result in permanent vision loss. The molecular mechanisms regulating neovascularization involve a delicate balance between proangiogenic and antiangiogenic factors^130^. When this balance tips toward proangiogenic stimuli, pathological neovascularization can occur. Among the key molecular players, vascular endothelial growth factor (VEGF) has been identified as a central mediator of pathological neovascularization^131^. Consequently, anti-VEGF therapies, including aflibercept, bevacizumab, and ranibizumab, have emerged as a standard of care in the management of neovascularization-associated diseases. However, anti-VEGF therapy is not uniformly efficacious and often necessitates repetitive intraocular injections for a lifetime, carrying a risk of endophthalmitis and posing a financial burden on patients. Consequently, the development of alternative or complementary therapeutic strategies have been pursued to sustain human vision. Due to the potential long-lasting effects, genome-editing therapy with CRISPR‒Cas9 has emerged as an alternative approach to treat chronic retinal and choroidal vascular disease.

In this context, in several studies, CRISPR‒Cas9 technologies have been used to knockout *Vegfa* in AMD- or choroidal-neovascularization mouse models. For example, in one study, *Vegfa* in the mouse RPE was targeted with lentiviral vectors carrying SpCas9 and sgRNA, achieving an indel formation efficacy of up to 84%^132^. In another study, LbCpf1, a CRISPR RNA-guided endonuclease derived from the *Lanchnospiraceae bacterium*, was delivered to the mouse retina using an AAV serotype-9 vector^133^. This approach led to indels in *Vegfa* with frequencies of 57.2% and 6.5% in the retina and RPE, respectively. Moreover, the knockout of *Vegfa* with AAV-LbCpf1-*Vegfa* reduced VEGFA levels in the RPE by 17 pg/mg and diminished the choroidal neovascularization (CNV) area by 42% in a laser-induced AMD mouse model. Aflibercept injection in the same model reduced the CNV area by 39%, indicating that the antiangiogenic effect of genome editing was comparable to that of aflibercept. Moreover, unlike aflibercept, the genome-editing approach demonstrated a long-term therapeutic effect even with a single intravitreal injection^133^.

Although AAV-mediated delivery of Cas9 has shown therapeutic efficacy, it can result in prolonged Cas9 expression and off-target edits. To address these concerns, a strategy involving transient Cas9 exposure in the form of mRNA or ribonucleoprotein (RNP) complexes has been developed. For instance, a recently developed lentiviral system, known as mLP-CRISPR, can achieve transient Cas9 expression by delivering a SpCas9 mRNA and sgRNA cassette co-packaged into the same viral particle^134^. This system transduced approximately two-thirds of RPE cells without transducing retinal cells^134^. A single subretinal injection of mLP-CRISPR targeting *Vegfa* knocked out 44% of *Vegfa* in the RPE and reduced the CNV area by 63% in a laser-induced AMD mouse model. Another study assessed the direct delivery of an RNP complex containing SpCas9 and sgRNA to knockout *Vegfa* in the RPE of a laser-induced AMD mouse model. A single subretinal injection of this RNP complex formed indels in *Vegfa* with a frequency of 22% and diminished the CNV area by 58% compared to a control RNP in the mouse model^135^.

In a recent study, the efficacy of sgRNA and paired gRNAs targeting *Vegfa* was examined in a laser-induced AMD mouse model^136^. The rationale behind using paired gRNAs was to enhance the efficiency of *Vegfa* disruption and CNV suppression. The selected sgRNAs targeted conserved regions in *Vegfa* across humans, rhesus macaques, and mice. Paired gRNAs demonstrated a higher rate of *Vegfa* disruption in vitro than sgRNA. However, paired gRNAs did not improve *Vegfa* disruption or reduce the CNV area in the mouse model. Taken together, these findings show the promise of CRISPR‒Cas9-mediated genome editing as an alternative or complementary therapy for neovascularization-associated diseases. Various strategies, including the use of different nucleases, delivery methods, and sgRNAs, have demonstrated efficient *Vegfa* knockout and a reduction in the CNV area.

### Inherited retinal diseases

IRDs encompass a diverse group of genetic diseases that lead to progressive vision loss or even blindness. Since the identification of a mutation causing an IRD in 1988^137^, over 270 genes responsible for IRDs have been identified and mapped to date^138,139^. Numerous institutes continue to focus on identifying the genetic causes of retinal diseases in patients without identifiable mutations. IRDs are characterized by a wide range of clinical presentations, including variable onset, severity, topography of retinal involvement, rate of progression, and mode of inheritance^140^. Historically, the management of most forms of IRDs has been largely symptomatic, but advances in understanding genetics and pathogenesis, along with technological developments, now offer various therapeutic opportunities^141^. Among these, gene-augmentation therapy for LCA caused by mutations in *RPE65* serves as the best example of treatment success^78^. Furthermore, the advent of genome editing offers new possibilities for treating a variety of IRDs, including retinitis pigmentosa (RP), Stargardt disease (STGD1), and LCA^13–15^.

#### Retinitis pigmentosa

RP represents a heterogeneous group of inherited retinal disorders characterized by the progressive degeneration of rod and cone photoreceptors, eventually leading to vision loss. RP is a leading cause of inherited visual impairment, with a worldwide prevalence of 1:4000^142^. To date, mutations in more than 80 genes have been identified in various RP subtypes with different patterns of inheritance, including autosomal-recessive, autosomal-dominant, and X-linked subtypes^142^. Despite numerous efforts to target RP, including nutritional therapy, gene therapy, retinal implants, and stem-cell therapy^143,144^, they are not yet successful due to low efficacy, poor durability, and concerns for safety^145^. For instance, gene-replacement strategies were found to compensate for loss-of-function mutations, but the treatment effects were only transient in mouse models. Additionally, these approaches are not applicable to autosomal-dominant RP (adRP)^146^.

Genome-editing tools have demonstrated potential in treating RP in various models. The Cas9-mediated NHEJ strategy is particularly suitable for adRP, as it enables specific disruption of the mutant allele while preserving the wild-type functional allele. By designing an sgRNA to specifically bind to the mutant allele and induce a DSB, NHEJ can lead to the introduction of mutations that disrupt the function of the mutant allele and alleviate the deleterious effect of the mutant protein. This approach showed promising results in the study of an adRP rat model, which carries the dominant S334ter mutation in the *Rho* gene (*Rho*^*S334*^). The *Rho* gene encodes a light-sensitive G protein-coupled receptor (GPCR), which plays a crucial role in vision by detecting light and initiating phototransduction. The S334ter mutation in the *Rho* gene introduces a premature stop codon, resulting in a truncated and nonfunctional protein (RHO^S334^). The RHO^S334^ protein lacks a signal sequence required for proper protein trafficking and prevents deactivation after light stimulation, thereby causing photoreceptor toxicity and apoptosis^147^. It was hypothesized that selective ablation of *Rho*^*S334*^ by Cas9-mediated NHEJ would eliminate RHO^S334^ production and toxicity. Subretinal injection of an sgRNA specific to the *Rho*^*S334*^ allele, along with the SpCas9 plasmid, led to specific disruption of *Rho*^*S334*^. This approach prevented retinal degeneration and improved visual function in a mouse model^147^.

The NHEJ strategy was also employed in a mouse model carrying the dominant P23H mutation in the *Rho* gene (*Rho*^*P23H*^), one of the most frequent adRP-associated mutations^148^. In animal studies, *Rho*^*P23H*^ was shown to cause gain-of-function pathological effects associated with protein misfolding and aggregation. The resulting P23H mutant protein was also shown to destabilize rod photoreceptor disk membranes and interfere with the process of disc membrane reorientation, causing photoreceptor toxicity^148^. To selectively ablate the pathogenic allele, an AAV carrying SpCas9 and *Rho*^*P23H*^-specific sgRNA was intravitreally injected into the mice. The treatment achieved a high rate of specific disruption of the mutant allele but not the wild-type allele, slowing photoreceptor degeneration and improving retinal function^148^.

Cas-mediated NHEJ proved useful in targeting the frameshift mutation in the Retinitis Pigmentosa GTPase Regulator (*RPGR*) gene, which causes the X-linked form of RP (XLRP)^149^. The RPGR protein is found in connecting cilia of photoreceptors, where it regulates the transport of various proteins and vesicles necessary for photoreceptor survival and function^149^. Mutations that disrupt the function of RPGR can impair the transport of proteins and cell homeostasis, leading to photoreceptor death^149^. To test whether NHEJ can treat XLRP caused by *RPGR* frameshift mutations, an AAV carrying SpCas9 and sgRNA was subretinally injected into a mouse model, which carries a frameshift mutation in *Rpgr*. The treatment restored the reading frame of the mutant *RPGR* in the mouse retina and alleviated the disease phenotype in mice^149^.

In contrast to NHEJ, Cas9-mediated HDR has been used in an RP mouse model to correct a point mutation in *Pde6b*, which accounts for 4 to 5% of autosomal-recessive RP cases^150^. The *Pde6b* gene encodes an enzyme called guanosine 3’,5’-monophosphate (cGMP) phosphodiesterase 6b (PDE6B), which plays a critical role in the visual signal-transduction pathway in photoreceptor cells. After phototransduction occurs, PDE6B breaks down cGMP in the outer segment of photoreceptor cells, returning the photoreceptor cells to their resting state. However, mutations in the *Pde6b* gene can lead to an accumulation of cGMP in photoreceptor cells, causing cellular stress and, ultimately, photoreceptor cell death. In one study, SpCas9, sgRNA, and a single-stranded donor template were delivered to a mouse model carrying the Y347X mutation in the *Pde6b* gene to correct the mutation. The treatment led to the restoration of wild-type levels of PDE6B by approximately 2%, resulting in improved photoreceptor survival and visual function. Later, prime editors enabled the correction of a different *Pde6b* point mutation (R560C) with much higher efficacy and precision. Dual-AAV-mediated delivery of the prime editor resulted in up to 76% correction of the mutation in mouse retinal cells, with indels no higher than 0.14%^151^. The treated mice exhibited restoration of PDE6B activity, preservation of photoreceptors, and an improvement in visual function^151^. Collectively, these novel therapeutic approaches offer considerable potential for tackling the challenges associated with RP and other IRDs. As these technologies continue to be refined, the prospects for safe, effective, and targeted RP treatments are likely to gain momentum, ultimately integrating genome editing into clinical practice.

#### Stargardt diseases

STGD1 is the most prevalent form of juvenile IRD, leading to progressive loss of central vision^152–154^. STGD1 is caused by autosomal-recessive mutations in the ATP Binding Cassette Subfamily A Member 4 (*ABCA4*) gene, which encodes the membrane transporter ABCA4^155^. This transporter facilitates the removal of all-trans-retinal (atRAL) from the photoreceptor outer segment of disc membranes as part of the visual cycle^156^. Mutations that impair ABCA4 function consequently lead to an accumulation of atRAL, which then forms condensation byproducts (A2E) in the lumen of disc membranes^157–159^. The build-up of atRAL and A2E is cytotoxic, accelerating the degeneration of photoreceptors and the RPE^157,160,161^. Despite its high prevalence, no treatment currently exists for STGD1. While gene-augmentation therapy using AAV has emerged as a promising therapy for IRDs, the 4.7-kb gene capacity of the AAV vector has limited treatment options for mutations in larger genes such as *ABCA4*^162^. This limitation precluded the possibility of AAV-based STGD1 gene therapy until the recent introduction of CRISPR‒Cas9 technology.

Several deep-intronic variants (DIVs) in *ABCA4* have been identified as causative for STGD1^163,164^. Despite their positions outside of the canonical exon, these genetic alterations can lead to aberrant splicing, the activation of cryptic splice sites, or the disruption of regulatory elements^165–167^. In a recent study, induced pluripotent stem cells (iPSCs) were generated from a patient with ABCA4 DIV c.5197-557G>T, which induces the retention of a 188-bp pseudoexon in the mature mRNA^168^. This pseudoexon leads to a frameshift in the open reading frame and the formation of a premature stop codon. Three approaches were employed, including SpCas9 with either sgRNA or paired gRNAs and SpCas9 nickase, to remove the DIV site in cone photoreceptor precursor cells (CPCs) differentiated from the patient iPSCs. Among these approaches, SpCas9 with sgRNA achieved the highest rescue of correct splicing with 83% and a 1.8-fold increase in the *ABCA4* transcript levels compared to untreated CPCs^168^. This result provided the first evidence of permanent splicing correction for STGD1 and demonstrated the potential of genome editing for the treatment of SGTD1 caused by DIVs.

#### Leber congenital amaurosis

LCA is a severe retinal dystrophy that manifests at birth or during early infancy, often resulting in progressive vision loss. Most patients with LCA experience severe visual impairment throughout childhood and become legally blind by the third or fourth decade of life^169^. LCA is clinically characterized by nystagmus, sluggish pupillary responses, night-blindness, and severely reduced or absent electroretinogram (ERG) signals^170^. Genetic studies have identified more than 20 genes associated with LCA, which encode proteins with diverse functions in photoreceptor development, maintenance, and function^169^. Until recently, no treatment has been available for LCA. However, the FDA-approved gene-augmentation therapy Luxturna has emerged as a targeted treatment option for LCA patients with biallelic mutations in *RPE65*^78,171,172^. In addition to gene-augmentation therapy, genome-editing technologies offer potential therapeutic strategies, having demonstrated therapeutic efficacy in mouse models of LCA subtypes.

The centrosomal protein 290 (*CEP290*) gene encodes a centrosomal protein that plays a crucial role in ciliogenesis, particularly in the formation and function of cilia. In photoreceptors, CEP290 is primarily localized in the connecting cilium, where it plays a critical role in cilium assembly and ciliary protein trafficking^173^. Mutations in *CEP290* can result in a subtype of LCA known as LCA10. The most common mutation is DIV c.2991+1655A<G, which generates a cryptic splice leading to the inclusion of an additional 128-bp cryptic exon with a premature stop codon^174–176^. To remove the DIV and restore normal splicing between exons 26 and 27 in a humanized LCA10 mouse model, SpCas9 and paired gRNAs were employed^177^. Subretinal injection of AAV carrying SpCas9 and paired gRNAs led to increased expression of wild-type CEP290 and decreased expression of defective CEP290. This successful preclinical study laid the foundation for initiating a clinical trial to treat LCA10 patients (NCT#03872479), and it highlights the potential of genome-editing technology in advancing therapies for LCA10.

Genome editing has been applied in the treatment of another subtype of LCA known as RPE65-associated LCA or LCA2. The *RPE65* gene, which is primarily expressed in the RPE, encodes an enzyme called the RPE-specific 65-kDa protein (RPE65). RPE65 plays a crucial role in the visual cycle by converting retinyl esters to 11-*cis*-retinol, a critical intermediate that is essential for the regeneration of visual chromophores and the phototransduction cascade in the retina^178^. Biallelic loss-of-function mutations in *RPE65* therefore disrupt the visual cycle and lead to impaired phototransduction and retinal degeneration. In 2017, the FDA-approved *RPE65* gene-replacement therapy, which delivers a normal cDNA copy of *RPE65*, as the first gene therapy for an inherited retinal disease. Although the therapy initially showed some improvement in the visual function of the patients, the extent and duration of the efficacy remain uncertain.

Cas9-mediated HDR was demonstrated as a new therapeutic strategy in a mouse model carrying a nonsense mutation (T to C) in *Rpe65*, also referred to as the *rd12* model^179^. The *rd12* model mice express a truncated, nonfunctional RPE65 protein, resulting in substantially impaired vision and early-onset photoreceptor cell death. SpCas9, gRNA, and donor template were delivered to the RPE of the mice using a dual-AAV approach^179^. However, this approach resulted in a low correction efficiency of approximately 1% and a higher-than-usual proportion of indel formation, making this approach suboptimal for therapeutic use^179^. In a later study, an adenine base editor was delivered to the same model mice using a lentiviral vector, resulting in a substantially higher correction efficiency of up to 27%, with less than 0.5% indel formation^48^. The treated mice showed significant improvement in their visual function, allowing them to discriminate direction, size, contrast, and spatial and temporal frequency^48^. Furthermore, selecting the optimal base editor variant could improve correction efficiency even more. For instance, the NG-ABE variant corrected up to 40% of *Rpe65* mRNA transcripts^51^. With the promising therapeutic effects of base editors, nonviral delivery approaches have been explored to further increase the safety of base editor delivery. For example, a lipofectamine-mediated delivery system was used to deliver the base editor and gRNA ribonucleoprotein complex to *rd12* mice, resulting in a correction efficiency of up to 5.7%^180^.

A recent study demonstrated the in vivo application of prime editing to correct a mutation in *rd12* mice^181^. Dual-AAV delivery of a prime editor and pegRNA resulted in the correction of approximately 28% of mutant alleles in transduced RPE. While the correction efficiency was not superior to that of a base editor, the rate of unintended edits, including substitutions and indels, was significantly lower. This feature makes prime editing particularly well suited for precise corrections in cases where bystander editing could not be tolerated or could have adverse effects.

## Current limitations and methods in development

### Immunogenic responses

Conventional gene therapy and genome-editing technologies have demonstrated significant potential for treating various ocular diseases. However, these approaches can provoke immunogenic responses, including both innate and adaptive immune responses, potentially compromising their safety and efficacy^182^. Although the eye is generally considered an immune-privileged organ, cases of ocular gene therapy leading to uveitis have been reported. Specifically, the use of AAV, commonly employed in gene therapy, can trigger immune responses and toxicity in the RPE and photoreceptors^182,183^. In several studies, it has been reported that a backflow of AAV into the vitreous following a subretinal injection can cause ocular inflammation in a dose-dependent manner^79,184,185^. As a result, various strategies are being explored to address the immunogenicity associated with AAV. One approach involves engineering capsid variants of AAV with reduced immunogenicity and enhanced transduction^186^. Another strategy incorporates short DNA oligonucleotides that antagonize Toll-like receptor 9 activation directly into the vector genome^187^.

Building on these findings, in a recent study, the presence of Cas9-reactive antibodies in serum and vitreous fluid samples from adult human subjects and mice was assessed^188^. The results revealed a high prevalence of preexisting antibodies against SpCas9 and *Staphylococcus aureus* Cas9 (SaCas9) in serum but not in the eye, suggesting a lower risk of immune responses in human eyes. However, a subset of mice developed antibodies against SpCas9 in the vitreous fluid following intraocular infection with *Streptococcus pyogenes*. These findings emphasize the need for further investigation to determine whether intraocular Cas9 exposure could elevate the risk of inflammation.

### Persistent expression of genome-editing tools

A critical concern in genome-editing therapies is the persistent expression of genome-editing tools, which can increase the risk of off-target effects and unanticipated consequences^13^. Ideally, these tools should remain active for only a limited duration, sufficient to achieve the desired genetic alterations, and then be degraded or become inactive to minimize adverse effects. Furthermore, viral delivery introduces the risk of viral DNA integration into the host genome, potentially increasing the risk of oncogenesis^189,190^. Additionally, persistent expression of the editing machinery can elicit long-term antiviral immune responses. Therefore, it is essential to develop strategies that limit the duration of genome-editing tool expression to the necessary timeframe without DNA integration, balancing therapeutic outcomes with minimized risks.

To address the persistent expression of genome-editing tools, nonviral delivery methods such as lipid nanoparticles, RNPs, and engineered viral-like particles have been developed^191–194^. These nonviral delivery systems can facilitate the transient expression of genome-editing tools and eliminate the risk of viral DNA integration. Additionally, these delivery systems are expected to exhibit less immunogenicity than viral vectors, further contributing to their potential as safer and more effective delivery methods for ocular genome editing.

### Suprachoroidal injection as a novel modality for delivering genome-editing tools to the retinal pigment epithelium and retina

The suprachoroidal space (SCS) is a potential space between the choroid and sclera. The choroid is highly vascular tissue comprised of unfenestrated endothelium responsible for oxygenation of the outer retina and RPE. The suprascleral space is advantageous for drug delivery because it offers a larger volume reservoir and is more easily accessible than the subretinal space. Suprachoroidal injection can promote panretinal delivery and sustained administration of therapeutic agents or genetic material more easily than subretinal injection^195^. However, there are challenges to delivering therapeutic agents from the suprachoroidal space to the retina because of Bruch’s membrane and the RPE^196^. Suprachoroidal injection of triamcinolone acetonide (TA), a glucocorticoid agonist is enormously successful in delivering steroid to the retina. Studies in rabbits in which suprachoroidal to intravitreal injections of TA were compared have demonstrated similar retinal concentrations but 20-fold less aqueous humor exposure after suprachoroidal injection, reducing the risk of cataract or steroid-induced elevation of intraocular pressure^197^.

In fact, suprachoroidal delivery of TA is now approved by the FDA for the treatment of noninfectious uveitic macular edema^198^. Trial reports documented that over half of the patients demonstrated improved acuity by 70 ETDRS letters and displayed reduced central subfield thickness by 150 μm at week 24 compared to sham-injected controls after a single suprachoroidal injection of 4 mg TA. There was no increase in the incidence of cataracts, with steroid-induced IOP elevation occurring approximately 10% of the time. The TYBEE trial thus far has shown the benefit of combination therapy of intravitreal aflibercept plus suprachoroidal TA compared to intravitreal aflibercept alone^199^. Similar data were reported in the Tanzanite study comparing combination therapy to monotherapy as described above for central retinal vein occlusion^200^. Triamcinolone acetate via SCS injection is also being tested for the treatment of diabetic macular edema.

The success of triamcinolone suggests that the solubility and clearance of the drug are related to the vascular supply of the choriocapillaris. Thus, using a bevacizumab hydrogel polymer in the SCS, recent progress has been made in achieving prolonged therapy targeting vascular endothelial growth factor (VEGF), with effects lasting up to 6 months in rabbits^201^. Remarkably, SCS injection is efficient for transfection with multiple delivery systems. Shen et al. injected nanoparticles containing a VEGF-expression plasmid into the SCS of mice, causing subretinal neovascularization that progressed to subretinal fibrosis, similar to untreated neovascular AMD. This demonstration provided a mechanistic view, suggesting that targeting VEGF via the SCS could be more effective than intravitreal injections^202^. Additionally, the injection of rats, either into the subretinal space or the SCS, with AAV8 vectors expressing anti-VEGF Fab resulted in similar suppression of VEGF-induced vascular leakage^203^. RGX-314 (REGENXBIO, Rockville, MD, USA) is an AAV8 encoding a transgene for anti-VEGF Fab fragment. RGX-314 is currently being tested in a Phase II trial for the treatment of wet age-related macular degeneration (NCT05407636) whereby it is injected into the subretinal space. The treatment comparator for this trial is intravitreal injection of aflibercept every 8 weeks. It is reasonable that therapeutics for neovascular and non-neovascular AMD delivered to the SCS might reach the retinal-RPE interface more readily than those delivered via intravitreal injection.

Current methods to deliver drug to the SCS include free-hand injection through the sclera, guarded microneedle injection, and tunneled microcatheters^204,205^. Commercially available guarded microcatheters have also been successfully employed in the CLS-TA trial (Clearside Biomedical, Alpharetta, GA, USA)^198^.

In summary, the choroid is a critical vascular source for the neural retina, and the SCS can be utilized for efficient therapeutic delivery of either small molecules or large viral vectors for gene transfer with the goal of treating severe neovascularization or providing genomic material for panretinal expression of genes necessary for vision.

## Concluding remarks

Genome-engineering technologies provide remarkable opportunities to advance the treatment of various ocular diseases. These cutting-edge tools allow the precise manipulation of genetic elements, enabling members of the scientific community and ophthalmologists to gain a deeper understanding of the molecular basis of ocular diseases and explore novel therapeutic approaches. The findings of preclinical studies have showcased the versatility of genome-engineering techniques in addressing ocular diseases with diverse genetic or nongenetic backgrounds. By targeting specific genes or regulatory regions, these studies have successfully corrected or mitigated disease phenotypes in animal models, establishing a foundation for more tailored and efficient treatments. Furthermore, the application of genome-editing techniques in ocular disease models has broadened our understanding of disease pathogenesis, potentially revealing new therapeutic targets and strategies.

As the field progresses, the incorporation of genome editing into clinical practice will require the development of standardized procedures, comprehensive assessment of safety and efficacy, and thorough long-term follow-up studies to achieve regulatory approval. With the ongoing evolution of our knowledge of ocular disease mechanisms and continuous advancements in genome-engineering technologies, we can anticipate the emergence of innovative, targeted, and potentially curative therapies. These groundbreaking treatments hold the promise of improving patient outcomes and quality of life, ultimately revolutionizing the management of ocular diseases.

## References

[1] Porteus MH (2019). A new class of medicines through DNA editing. N. Engl. J. Med..

[2] Anzalone AV, Koblan LW, Liu DR (2020). Genome editing with CRISPR-Cas nucleases, base editors, transposases and prime editors. Nat. Biotechnol..

[3] Jinek M (2012). A programmable dual-RNA-guided DNA endonuclease in adaptive bacterial immunity. Science.

[4] Doudna JA, Charpentier E (2014). Genome editing. The new frontier of genome engineering with CRISPR-Cas9. Science.

[5] Wang JY, Doudna JA (2023). CRISPR technology: a decade of genome editing is only the beginning. Science.

[6] Komor AC, Kim YB, Packer MS, Zuris JA, Liu DR (2016). Programmable editing of a target base in genomic DNA without double-stranded DNA cleavage. Nature.

[7] Gaudelli NM (2017). Programmable base editing of A•T to G•C in genomic DNA without DNA cleavage. Nature.

[8] Anzalone AV (2019). Search-and-replace genome editing without double-strand breaks or donor DNA. Nature.

[9] Newby GA, Liu DR (2021). In vivo somatic cell base editing and prime editing. Mol. Ther..

[10] Rees HA, Liu DR (2018). Base editing: precision chemistry on the genome and transcriptome of living cells. Nat. Rev. Genet..

[11] Chen PJ, Liu DR (2023). Prime editing for precise and highly versatile genome manipulation. Nat. Rev. Genet..

[12] Caruso, S. M., Quinn, P. M., da Costa, B. L. & Tsang, S. H. CRISPR/Cas therapeutic strategies for autosomal dominant disorders. *J. Clin. Invest.* **132**, e158287 (2022). 10.1172/JCI158287 PMC9057583 35499084

[13] Suh S, Choi EH, Raguram A, Liu DR, Palczewski K (2022). Precision genome editing in the eye. Proc. Natl Acad. Sci. USA.

[14] Yan AL, Du SW, Palczewski K (2023). Genome editing, a superior therapy for inherited retinal diseases. Vis. Res..

[15] Du, S. W. & Palczewski, K. Eye on genome editing. *J. Exp. Med.* **220**, e20230146 (2023). 10.1084/jem.20230146 PMC10035435 36930175

[16] Palczewska G (2010). Noninvasive multiphoton fluorescence microscopy resolves retinol and retinal condensation products in mouse eyes. Nat. Med..

[17] Palczewska G (2014). Noninvasive two-photon microscopy imaging of mouse retina and retinal pigment epithelium through the pupil of the eye. Nat. Med..

[18] Palczewska G (2020). Noninvasive two-photon optical biopsy of retinal fluorophores. Proc. Natl Acad. Sci. USA.

[19] Boguslawski, J. et al. In vivo imaging of the human eye using a 2-photon-excited fluorescence scanning laser ophthalmoscope. *J. Clin. Invest.* **132**, e154218 (2022). 10.1172/JCI154218 PMC8759795 34847075

[20] Palczewska G, Wojtkowski M, Palczewski K (2023). From mouse to human: accessing the biochemistry of vision in vivo by two-photon excitation. Prog. Retin. Eye Res..

[21] Ishino Y, Shinagawa H, Makino K, Amemura M, Nakata A (1987). Nucleotide sequence of the iap gene, responsible for alkaline phosphatase isozyme conversion in *Escherichia coli*, and identification of the gene product. J. Bacteriol..

[22] Mojica FJ, Díez-Villaseñor C, Soria E, Juez G (2000). Biological significance of a family of regularly spaced repeats in the genomes of Archaea, Bacteria and mitochondria. Mol. Microbiol..

[23] Jansen R, Embden JD, Gaastra W, Schouls LM (2002). Identification of genes that are associated with DNA repeats in prokaryotes. Mol. Microbiol..

[24] Mojica FJ, Diez-Villasenor C, Garcia-Martinez J, Soria E (2005). Intervening sequences of regularly spaced prokaryotic repeats derive from foreign genetic elements. J. Mol. Evol..

[25] Bolotin A, Quinquis B, Sorokin A, Ehrlich SD (2005). Clustered regularly interspaced short palindrome repeats (CRISPRs) have spacers of extrachromosomal origin. Microbiology.

[26] Pourcel C, Salvignol G, Vergnaud G (2005). CRISPR elements in *Yersinia pestis* acquire new repeats by preferential uptake of bacteriophage DNA, and provide additional tools for evolutionary studies. Microbiology.

[27] Barrangou R (2007). CRISPR provides acquired resistance against viruses in prokaryotes. Science.

[28] Makarova KS, Grishin NV, Shabalina SA, Wolf YI, Koonin EV (2006). A putative RNA-interference-based immune system in prokaryotes: computational analysis of the predicted enzymatic machinery, functional analogies with eukaryotic RNAi, and hypothetical mechanisms of action. Biol. Direct.

[29] Makarova KS (2011). Evolution and classification of the CRISPR–Cas systems. Nat. Rev. Microbiol..

[30] van der Oost J, Westra ER, Jackson RN, Wiedenheft B (2014). Unravelling the structural and mechanistic basis of CRISPR–Cas systems. Nat. Rev. Microbiol..

[31] Gasiunas G, Barrangou R, Horvath P, Siksnys V (2012). Cas9-crRNA ribonucleoprotein complex mediates specific DNA cleavage for adaptive immunity in bacteria. Proc. Natl Acad. Sci. USA.

[32] Ceccaldi R, Rondinelli B, D’Andrea AD (2016). Repair pathway choices and consequences at the double-strand break. Trends Cell Biol..

[33] Carroll D (2014). Genome engineering with targetable nucleases. Annu. Rev. Biochem..

[34] Doudna JA, Charpentier E (2014). The new frontier of genome engineering with CRISPR-Cas9. Science.

[35] Maruyama T (2015). Increasing the efficiency of precise genome editing with CRISPR-Cas9 by inhibition of nonhomologous end joining. Nat. Biotechnol..

[36] Cong L (2013). Multiplex genome engineering using CRISPR/Cas systems. Science.

[37] Musunuru K (2021). In vivo CRISPR base editing of PCSK9 durably lowers cholesterol in primates. Nature.

[38] Koblan LW (2018). Improving cytidine and adenine base editors by expression optimization and ancestral reconstruction. Nat. Biotechnol..

[39] Anzalone AV, Koblan LW, Liu DR (2020). Genome editing with CRISPR–Cas nucleases, base editors, transposases and prime editors. Nat. Biotechnol..

[40] Lin Q (2020). Prime genome editing in rice and wheat. Nat. Biotechnol..

[41] Nishimasu H (2014). Crystal structure of Cas9 in complex with guide RNA and target DNA. Cell.

[42] Jiang F, Doudna JA (2017). CRISPR–Cas9 structures and mechanisms. Annu. Rev. Biophys..

[43] Nishida, K. et al. Targeted nucleotide editing using hybrid prokaryotic and vertebrate adaptive immune systems. *Science* **353**, aaf8729 (2016). 10.1126/science.aaf8729 27492474

[44] Li X (2018). Base editing with a Cpf1-cytidine deaminase fusion. Nat. Biotechnol..

[45] Song Y (2020). Large-fragment deletions induced by Cas9 cleavage while not in the BEs system. Mol. Ther. Nucleic Acids.

[46] Komor AC, Badran AH, Liu DR (2017). CRISPR-based technologies for the manipulation of eukaryotic genomes. Cell.

[47] Ryu S-M (2018). Adenine base editing in mouse embryos and an adult mouse model of Duchenne muscular dystrophy. Nat. Biotechnol..

[48] Suh S (2021). Restoration of visual function in adult mice with an inherited retinal disease via adenine base editing. Nat. Biomed. Eng..

[49] Koblan LW (2021). In vivo base editing rescues Hutchinson–Gilford progeria syndrome in mice. Nature.

[50] Newby GA (2021). Base editing of haematopoietic stem cells rescues sickle cell disease in mice. Nature.

[51] Choi EH (2022). In vivo base editing rescues cone photoreceptors in a mouse model of early-onset inherited retinal degeneration. Nat. Commun..

[52] Reichart D (2023). Efficient in vivo genome editing prevents hypertrophic cardiomyopathy in mice. Nat. Med..

[53] Komor AC (2017). Improved base excision repair inhibition and bacteriophage Mu Gam protein yields C:G-to-T:A base editors with higher efficiency and product purity. Sci. Adv..

[54] Zafra MP (2018). Optimized base editors enable efficient editing in cells, organoids and mice. Nat. Biotechnol..

[55] Richter MF (2020). Phage-assisted evolution of an adenine base editor with improved Cas domain compatibility and activity. Nat. Biotechnol..

[56] Miller SM, Wang T, Liu DR (2020). Phage-assisted continuous and non-continuous evolution. Nat. Protoc..

[57] Gaudelli NM (2020). Directed evolution of adenine base editors with increased activity and therapeutic application. Nat. Biotechnol..

[58] Jin S (2019). Cytosine, but not adenine, base editors induce genome-wide off-target mutations in rice. Science.

[59] Zuo E (2019). Cytosine base editor generates substantial off-target single-nucleotide variants in mouse embryos. Science.

[60] Gao R (2022). Genomic and transcriptomic analyses of prime editing guide RNA–independent off-target effects by prime editors. CRISPR J..

[61] Park S-J (2021). Targeted mutagenesis in mouse cells and embryos using an enhanced prime editor. Genome Biol..

[62] Velimirovic M (2022). Peptide fusion improves prime editing efficiency. Nat. Commun..

[63] Song M (2021). Generation of a more efficient prime editor 2 by addition of the Rad51 DNA-binding domain. Nat. Commun..

[64] Nelson JW (2022). Engineered pegRNAs improve prime editing efficiency. Nat. Biotechnol..

[65] Zhang G (2022). Enhancement of prime editing via xrRNA motif-joined pegRNA. Nat. Commun..

[66] Anzalone AV (2022). Programmable deletion, replacement, integration and inversion of large DNA sequences with twin prime editing. Nat. Biotechnol..

[67] Liu N (2022). HDAC inhibitors improve CRISPR-Cas9 mediated prime editing and base editing. Mol. Ther. Nucleic Acids.

[68] Chen PJ (2021). Enhanced prime editing systems by manipulating cellular determinants of editing outcomes. Cell.

[69] Kevany BM, Palczewski K (2010). Phagocytosis of retinal rod and cone photoreceptors. Physiology.

[70] Kiser PD, Palczewski K (2016). Retinoids and retinal diseases. Annu. Rev. Vis. Sci..

[71] Flaxman SR (2017). Global causes of blindness and distance vision impairment 1990-2020: a systematic review and meta-analysis. Lancet Glob. Health.

[72] Taylor A (2009). Ocular immune privilege. Eye.

[73] Streilein JW (2003). Ocular immune privilege: therapeutic opportunities from an experiment of nature. Nat. Rev. Immunol..

[74] Sapino, S. et al. Ocular drug delivery: a special focus on the thermosensitive approach. *Nanomaterials* **9**, 884 (2019). 10.3390/nano9060884 PMC6630567 31207951

[75] Dias MF (2018). Molecular genetics and emerging therapies for retinitis pigmentosa: basic research and clinical perspectives. Prog. Retin. Eye Res..

[76] Kumar S (2023). RNA-targeting strategies as a platform for ocular gene therapy. Prog. Retin. Eye Res..

[77] Jo, D. H., Bae, S., Kim, H. H., Kim, J. S. & Kim, J. H. In vivo application of base and prime editing to treat inherited retinal diseases. *Prog. Retin. Eye Res.* **94**, 101132 (2023). 10.1016/j.preteyeres.2022.101132 36241547

[78] Russell S (2017). Efficacy and safety of voretigene neparvovec (AAV2-hRPE65v2) in patients with RPE65-mediated inherited retinal dystrophy: a randomised, controlled, open-label, phase 3 trial. Lancet.

[79] Bainbridge JW (2015). Long-term effect of gene therapy on Leber’s congenital amaurosis. N. Engl. J. Med..

[80] Jacobson SG (2015). Improvement and decline in vision with gene therapy in childhood blindness. N. Engl. J. Med..

[81] Cideciyan AV (2013). Human retinal gene therapy for Leber congenital amaurosis shows advancing retinal degeneration despite enduring visual improvement. Proc. Natl Acad. Sci. USA.

[82] Gardiner KL (2020). Long-term structural outcomes of late-stage RPE65 gene therapy. Mol. Ther..

[83] Eghrari, A. O., Riazuddin, S. A. & Gottsch, J. D. Overview of the cornea: structure, function, and development. *Prog. Mol. Biol. Transl. Sci*. **134**, 7–23 (2015). 10.1016/bs.pmbts.2015.04.001 26310146

[84] Meek KM, Dennis S, Khan S (2003). Changes in the refractive index of the stroma and its extrafibrillar matrix when the cornea swells. Biophys. J..

[85] Liesegang TJ (2001). Herpes simplex virus epidemiology and ocular importance. Cornea.

[86] Iliff BW, Riazuddin SA, Gottsch JD (2012). The genetics of Fuchs’ corneal dystrophy. Expert Rev. Ophthalmol..

[87] Munier FL (1997). Kerato-epithelin mutations in four 5q31-linked corneal dystrophies. Nat. Genet..

[88] Farooq AV, Shukla D (2012). Herpes simplex epithelial and stromal keratitis: an epidemiologic update. Surv. Ophthalmol..

[89] Weerasooriya S, DiScipio KA, Darwish AS, Bai P, Weller SK (2019). Herpes simplex virus 1 ICP8 mutant lacking annealing activity is deficient for viral DNA replication. Proc. Natl Acad. Sci. USA.

[90] Weller SK, Coen DM (2012). Herpes simplex viruses: mechanisms of DNA replication. Cold Spring Harb. Perspect. Biol..

[91] Yin D (2021). Targeting herpes simplex virus with CRISPR-Cas9 cures herpetic stromal keratitis in mice. Nat. Biotechnol..

[92] Wei, A. et al. In vivo CRISPR gene editing in patients with herpes stromal keratitis. Preprint at *medRxiv*10.1101/2023.02.21.23285822 (2023).

[93] Gottsch JD (2005). Inheritance of a novel COL8A2 mutation defines a distinct early-onset subtype of Fuchs corneal dystrophy. Invest. Ophthalmol. Vis. Sci..

[94] Biswas S (2001). Missense mutations in COL8A2, the gene encoding the alpha2 chain of type VIII collagen, cause two forms of corneal endothelial dystrophy. Hum. Mol. Genet..

[95] Jun AS (2012). An alpha 2 collagen VIII transgenic knock-in mouse model of Fuchs endothelial corneal dystrophy shows early endothelial cell unfolded protein response and apoptosis. Hum. Mol. Genet..

[96] Meng H (2013). L450W and Q455K Col8a2 knock-in mouse models of Fuchs endothelial corneal dystrophy show distinct phenotypes and evidence for altered autophagy. Invest. Ophthalmol. Vis. Sci..

[97] Uehara, H. et al. Start codon disruption with CRISPR/Cas9 prevents murine Fuchs’ endothelial corneal dystrophy. *Elife* **10**, e55637 (2021). 10.7554/eLife.55637 PMC8216720 34100716

[98] Lakshminarayanan R (2014). Clinical and genetic aspects of the TGFBI-associated corneal dystrophies. Ocul. Surf..

[99] Akiya S, Takahashi H, Nakano N, Hirose N, Tokuda Y (1999). Granular-lattice (Avellino) corneal dystrophy. Ophthalmologica.

[100] Folberg R (1988). Clinically atypical granular corneal dystrophy with pathologic features of lattice-like amyloid deposits. A study of these families. Ophthalmology.

[101] Moon JW (2007). Homozygous granular corneal dystrophy type II (Avellino corneal dystrophy): natural history and progression after treatment. Cornea.

[102] Watanabe H (2001). Two patterns of opacity in corneal dystrophy caused by the homozygous BIG-H3 R124H mutation. Am. J. Ophthalmol..

[103] Dinh R, Rapuano CJ, Cohen EJ, Laibson PR (1999). Recurrence of corneal dystrophy after excimer laser phototherapeutic keratectomy. Ophthalmology.

[104] Lyons CJ (1994). Granular corneal dystrophy. Visual results and pattern of recurrence after lamellar or penetrating keratoplasty. Ophthalmology.

[105] Taketani Y (2017). Repair of the TGFBI gene in human corneal keratocytes derived from a granular corneal dystrophy patient via CRISPR/Cas9-induced homology-directed repair. Sci. Rep..

[106] Weinreb RN, Aung T, Medeiros FA (2014). The pathophysiology and treatment of glaucoma: a review. JAMA.

[107] Tham YC (2014). Global prevalence of glaucoma and projections of glaucoma burden through 2040: a systematic review and meta-analysis. Ophthalmology.

[108] Weinreb RN, Khaw PT (2004). Primary open-angle glaucoma. Lancet.

[109] Stone EM (1997). Identification of a gene that causes primary open angle glaucoma. Science.

[110] Goldenstein H, Levy NS, Levy AP (2012). Haptoglobin genotype and its role in determining heme-iron mediated vascular disease. Pharmacol. Res..

[111] Kasetti, R. B. et al. Autophagy stimulation reduces ocular hypertension in a murine glaucoma model via autophagic degradation of mutant myocilin. *JCI Insight* **6**, e143359 (2021). 10.1172/jci.insight.143359 PMC8021112 33539326

[112] Jain A (2017). CRISPR-Cas9-based treatment of myocilin-associated glaucoma. Proc. Natl Acad. Sci. USA.

[113] Wu J (2020). Gene therapy for glaucoma by ciliary body aquaporin 1 disruption using CRISPR-Cas9. Mol. Ther..

[114] Derynck, R. & Budi, E. H. Specificity, versatility, and control of TGF-β family signaling. *Sci. Signal.* **12**, eaav5183 (2019). 10.1126/scisignal.aav5183 PMC6800142 30808818

[115] Cousins SW, McCabe MM, Danielpour D, Streilein JW (1991). Identification of transforming growth factor-beta as an immunosuppressive factor in aqueous humor. Invest. Ophthalmol. Vis. Sci..

[116] Granstein RD (1990). Aqueous humor contains transforming growth factor-beta and a small (less than 3500 daltons) inhibitor of thymocyte proliferation. J. Immunol..

[117] Jampel HD, Roche N, Stark WJ, Roberts AB (1990). Transforming growth factor-beta in human aqueous humor. Curr. Eye Res..

[118] Ozcan AA, Ozdemir N, Canataroglu A (2004). The aqueous levels of TGF-beta2 in patients with glaucoma. Int. Ophthalmol..

[119] Yamamoto N, Itonaga K, Marunouchi T, Majima K (2005). Concentration of transforming growth factor beta2 in aqueous humor. Ophthalmic Res..

[120] Trivedi RH, Nutaitis M, Vroman D, Crosson CE (2011). Influence of race and age on aqueous humor levels of transforming growth factor-beta 2 in glaucomatous and nonglaucomatous eyes. J. Ocul. Pharm. Ther..

[121] Pena JD, Taylor AW, Ricard CS, Vidal I, Hernandez MR (1999). Transforming growth factor beta isoforms in human optic nerve heads. Br. J. Ophthalmol..

[122] Rayana NP (2021). Using CRISPR interference as a therapeutic approach to treat TGFβ2-induced ocular hypertension and glaucoma. Invest. Ophthalmol. Vis. Sci..

[123] Gilbert LA (2013). CRISPR-mediated modular RNA-guided regulation of transcription in eukaryotes. Cell.

[124] Thakore PI (2015). Highly specific epigenome editing by CRISPR-Cas9 repressors for silencing of distal regulatory elements. Nat. Methods.

[125] Carmeliet P (2003). Angiogenesis in health and disease. Nat. Med..

[126] Wong TY, Cheung CM, Larsen M, Sharma S, Simó R (2016). Diabetic retinopathy. Nat. Rev. Dis. Prim..

[127] Hellström A, Smith LE, Dammann O (2013). Retinopathy of prematurity. Lancet.

[128] Jager RD, Mieler WF, Miller JW (2008). Age-related macular degeneration. N. Engl. J. Med..

[129] Guymer, R. H. & Campbell, T. G. Age-related macular degeneration. *Lancet* **401**, P1459–P1472 (2023). 10.1016/S0140-6736(22)02609-5 36996856

[130] Campochiaro PA (2015). Molecular pathogenesis of retinal and choroidal vascular diseases. Prog. Retin. Eye Res..

[131] Miller JW, Le Couter J, Strauss EC, Ferrara N (2013). Vascular endothelial growth factor A in intraocular vascular disease. Ophthalmology.

[132] Holmgaard A (2017). In vivo knockout of the *Vegfa* gene by lentiviral delivery of CRISPR/Cas9 in mouse retinal pigment epithelium cells. Mol. Ther. Nucleic Acids.

[133] Koo T (2018). CRISPR-LbCpf1 prevents choroidal neovascularization in a mouse model of age-related macular degeneration. Nat. Commun..

[134] Ling S (2021). Lentiviral delivery of co-packaged Cas9 mRNA and a Vegfa-targeting guide RNA prevents wet age-related macular degeneration in mice. Nat. Biomed. Eng..

[135] Kim K (2017). Genome surgery using Cas9 ribonucleoproteins for the treatment of age-related macular degeneration. Genome Res..

[136] Chung SH (2022). CRISPR-based VEGF suppression using paired guide RNAs for treatment of choroidal neovascularization. Mol. Ther. Nucleic Acids.

[137] Mitchell GA (1988). An initiator codon mutation in ornithine-delta-aminotransferase causing gyrate atrophy of the choroid and retina. J. Clin. Investig..

[138] Duncan JL (2018). Inherited retinal degenerations: current landscape and knowledge gaps. Transl. Vis. Sci. Technol..

[139] Thompson DA (2020). Advancing clinical trials for inherited retinal diseases: recommendations from the Second Monaciano Symposium. Transl. Vis. Sci. Technol..

[140] Georgiou M, Fujinami K, Michaelides M (2021). Inherited retinal diseases: therapeutics, clinical trials and end points—a review. Clin. Exp. Ophthalmol..

[141] Cideciyan AV (2021). Measures of function and structure to determine phenotypic features, natural history, and treatment outcomes in inherited retinal diseases. Annu. Rev. Vis. Sci..

[142] Verbakel SK (2018). Non-syndromic retinitis pigmentosa. Prog. Retin. Eye Res..

[143] Hosseini Shabanan S, Seyedmirzaei H, Barnea A, Hanaei S, Rezaei N (2022). Stem cell transplantation as a progressing treatment for retinitis pigmentosa. Cell Tissue Res..

[144] Stingl K (2015). Subretinal visual implant Alpha IMS—clinical trial interim report. Vis. Res..

[145] Merkle FT (2017). Human pluripotent stem cells recurrently acquire and expand dominant negative P53 mutations. Nature.

[146] Petrs-Silva H, Linden R (2014). Advances in gene therapy technologies to treat retinitis pigmentosa. Clin. Ophthalmol..

[147] Bakondi B (2016). In vivo CRISPR/Cas9 gene editing corrects retinal dystrophy in the S334ter-3 rat model of autosomal dominant retinitis pigmentosa. Mol. Ther..

[148] Giannelli SG (2018). Cas9/sgRNA selective targeting of the P23H Rhodopsin mutant allele for treating retinitis pigmentosa by intravitreal AAV9.PHP.B-based delivery. Hum. Mol. Genet..

[149] Gumerson JD (2022). Restoration of RPGR expression in vivo using CRISPR/Cas9 gene editing. Gene Ther..

[150] Cai Y (2019). In vivo genome editing rescues photoreceptor degeneration via a Cas9/RecA-mediated homology-directed repair pathway. Sci. Adv..

[151] Qin H (2023). Vision rescue via unconstrained in vivo prime editing in degenerating neural retinas. J. Exp. Med..

[152] Tanna P, Strauss RW, Fujinami K, Michaelides M (2017). Stargardt disease: clinical features, molecular genetics, animal models and therapeutic options. Br. J. Ophthalmol..

[153] Fujinami K (2015). Clinical and molecular characteristics of childhood-onset Stargardt disease. Ophthalmology.

[154] Molday LL, Rabin AR, Molday RS (2000). ABCR expression in foveal cone photoreceptors and its role in Stargardt macular dystrophy. Nat. Genet..

[155] Allikmets R (1997). A photoreceptor cell-specific ATP-binding transporter gene (ABCR) is mutated in recessive Stargardt macular dystrophy. Nat. Genet..

[156] Molday RS, Garces FA, Scortecci JF, Molday LL (2022). Structure and function of ABCA4 and its role in the visual cycle and Stargardt macular degeneration. Prog. Retin. Eye Res..

[157] Cideciyan AV (2004). Mutations in ABCA4 result in accumulation of lipofuscin before slowing of the retinoid cycle: a reappraisal of the human disease sequence. Hum. Mol. Genet..

[158] Beharry S, Zhong M, Molday RS (2004). N-retinylidene-phosphatidylethanolamine is the preferred retinoid substrate for the photoreceptor-specific ABC transporter ABCA4 (ABCR). J. Biol. Chem..

[159] Quazi F, Lenevich S, Molday RS (2012). ABCA4 is an N-retinylidene-phosphatidylethanolamine and phosphatidylethanolamine importer. Nat. Commun..

[160] Chen Y (2012). Mechanism of all-trans-retinal toxicity with implications for Stargardt disease and age-related macular degeneration. J. Biol. Chem..

[161] Weng J (1999). Insights into the function of Rim protein in photoreceptors and etiology of Stargardt’s disease from the phenotype in abcr knockout mice. Cell.

[162] Grieger JC, Samulski RJ (2005). Packaging capacity of adeno-associated virus serotypes: impact of larger genomes on infectivity and postentry steps. J. Virol..

[163] Zernant J (2014). Analysis of the ABCA4 genomic locus in Stargardt disease. Hum. Mol. Genet..

[164] Sangermano R (2018). ABCA4 midigenes reveal the full splice spectrum of all reported noncanonical splice site variants in Stargardt disease. Genome Res..

[165] Scotti MM, Swanson MS (2016). RNA mis-splicing in disease. Nat. Rev. Genet..

[166] Cartegni L, Chew SL, Krainer AR (2002). Listening to silence and understanding nonsense: exonic mutations that affect splicing. Nat. Rev. Genet..

[167] Ong CT, Corces VG (2011). Enhancer function: new insights into the regulation of tissue-specific gene expression. Nat. Rev. Genet..

[168] De Angeli P (2022). Effective splicing restoration of a deep-intronic ABCA4 variant in cone photoreceptor precursor cells by CRISPR/SpCas9 approaches. Mol. Ther. Nucleic Acids.

[169] den Hollander AI, Roepman R, Koenekoop RK, Cremers FP (2008). Leber congenital amaurosis: genes, proteins and disease mechanisms. Prog. Retin. Eye Res..

[170] Kumaran N, Moore AT, Weleber RG, Michaelides M (2017). Leber congenital amaurosis/early-onset severe retinal dystrophy: clinical features, molecular genetics and therapeutic interventions. Br. J. Ophthalmol..

[171] Bennett J (2016). Safety and durability of effect of contralateral-eye administration of AAV2 gene therapy in patients with childhood-onset blindness caused by RPE65 mutations: a follow-on phase 1 trial. Lancet.

[172] Maguire AM (2019). Efficacy, safety, and durability of voretigene neparvovec-rzyl in RPE65 mutation-associated inherited retinal dystrophy: results of phase 1 and 3 trials. Ophthalmology.

[173] Rachel RA, Li T, Swaroop A (2012). Photoreceptor sensory cilia and ciliopathies: focus on CEP290, RPGR and their interacting proteins. Cilia.

[174] Perrault I (2007). Spectrum of NPHP6/CEP290 mutations in Leber congenital amaurosis and delineation of the associated phenotype. Hum. Mutat..

[175] Vallespin E (2007). Frequency of CEP290 c.2991_1655A>G mutation in 175 Spanish families affected with Leber congenital amaurosis and early-onset retinitis pigmentosa. Mol. Vis..

[176] den Hollander AI (2006). Mutations in the CEP290 (NPHP6) gene are a frequent cause of Leber congenital amaurosis. Am. J. Hum. Genet..

[177] Maeder ML (2019). Development of a gene-editing approach to restore vision loss in Leber congenital amaurosis type 10. Nat. Med..

[178] Cai X, Conley SM, Naash MI (2009). RPE65: role in the visual cycle, human retinal disease, and gene therapy. Ophthalmic Genet..

[179] Jo DH (2019). CRISPR-Cas9-mediated therapeutic editing of Rpe65 ameliorates the disease phenotypes in a mouse model of Leber congenital amaurosis. Sci. Adv..

[180] Jang H-K (2021). High-purity production and precise editing of DNA base editing ribonucleoproteins. Sci. Adv..

[181] Jang H (2022). Application of prime editing to the correction of mutations and phenotypes in adult mice with liver and eye diseases. Nat. Biomed. Eng..

[182] Reichel FF (2017). AAV8 can induce innate and adaptive immune response in the primate eye. Mol. Ther..

[183] Xiong W (2019). AAV cis-regulatory sequences are correlated with ocular toxicity. Proc. Natl Acad. Sci. USA.

[184] Bainbridge JW (2008). Effect of gene therapy on visual function in Leber’s congenital amaurosis. N. Engl. J. Med..

[185] Dimopoulos IS (2018). Two-year results after AAV2-mediated gene therapy for choroideremia: the Alberta experience. Am. J. Ophthalmol..

[186] Dalkara D (2013). In vivo-directed evolution of a new adeno-associated virus for therapeutic outer retinal gene delivery from the vitreous. Sci. Transl. Med..

[187] Chan, Y. K. et al. Engineering adeno-associated viral vectors to evade innate immune and inflammatory responses. *Sci. Transl. Med.* **13**, eabd3438 (2021). 10.1126/scitranslmed.abd3438 PMC8409505 33568518

[188] Toral MA (2022). Investigation of Cas9 antibodies in the human eye. Nat. Commun..

[189] Chandler RJ, Sands MS, Venditti CP (2017). Recombinant adeno-associated viral integration and genotoxicity: insights from animal models. Hum. Gene Ther..

[190] Donsante A (2001). Observed incidence of tumorigenesis in long-term rodent studies of rAAV vectors. Gene Ther..

[191] Finn JD (2018). A single administration of CRISPR/Cas9 lipid nanoparticles achieves robust and persistent in vivo genome editing. Cell Rep..

[192] Lyu P, Javidi-Parsijani P, Atala A, Lu B (2019). Delivering Cas9/sgRNA ribonucleoprotein (RNP) by lentiviral capsid-based bionanoparticles for efficient ‘hit-and-run’ genome editing. Nucleic Acids Res..

[193] Mangeot PE (2019). Genome editing in primary cells and in vivo using viral-derived nanoblades loaded with Cas9-sgRNA ribonucleoproteins. Nat. Commun..

[194] Banskota S (2022). Engineered virus-like particles for efficient in vivo delivery of therapeutic proteins. Cell.

[195] Patel SR (2012). Targeted administration into the suprachoroidal space using a microneedle for drug delivery to the posterior segment of the eye. Invest. Ophthalmol. Vis. Sci..

[196] Olsen TW (2020). Drug tissue distribution of TUDCA from a biodegradable suprachoroidal implant versus intravitreal or systemic delivery in the pig model. Transl. Vis. Sci. Technol..

[197] Muya L, Kansara V, Cavet ME, Ciulla T (2022). Suprachoroidal injection of triamcinolone acetonide suspension: ocular pharmacokinetics and distribution in rabbits demonstrates high and durable levels in the chorioretina. J. Ocul. Pharm. Ther..

[198] Yeh S (2020). Efficacy and safety of suprachoroidal CLS-TA for macular edema secondary to noninfectious uveitis: phase 3 randomized trial. Ophthalmology.

[199] Barakat MR (2021). Suprachoroidal CLS-TA plus intravitreal aflibercept for diabetic macular edema: a randomized, double-masked, parallel-design, controlled study. Ophthalmol. Retin..

[200] Campochiaro PA (2018). Suprachoroidal triamcinolone acetonide for retinal vein occlusion: results of the tanzanite study. Ophthalmol. Retin..

[201] Jung JH, Kim SS, Chung H, Hejri A, Prausnitz MR (2022). Six-month sustained delivery of anti-VEGF from in-situ forming hydrogel in the suprachoroidal space. J. Control Release.

[202] Shen, J. et al. Suprachoroidal gene transfer with nonviral nanoparticles. *Sci. Adv.* **6**, eaba1606 (2020). 10.1126/sciadv.aba1606 PMC7458446 32937452

[203] Ding K (2019). AAV8-vectored suprachoroidal gene transfer produces widespread ocular transgene expression. J. Clin. Invest..

[204] Kansara VS (2020). Suprachoroidally delivered DNA nanoparticles transfect retina and retinal pigment epithelium/choroid in rabbits. Transl. Vis. Sci. Technol..

[205] Xu D, Khan MA, Ho AC (2021). Creating an ocular biofactory: surgical approaches in gene therapy for acquired retinal diseases. Asia Pac. J. Ophthalmol..

